# Predictive value of the residual cholesterol inflammation index for heart failure risk: A cross-sectional study from NHANES

**DOI:** 10.1097/MD.0000000000047575

**Published:** 2026-02-06

**Authors:** Ji Ouyang, Jiayuan Song, Xingxing Zhang, Guicheng Liu, Zhixi Hu

**Affiliations:** a College of traditional Chinese medicine, Hunan University of Traditional Chinese Medicine, Changsha, Hunan, China; b College of Integrative Medicine, Changchun University of Chinese Medicine, Changchun, Jilin, China.

**Keywords:** cross-sectional study, heart failure, NHANES, predictive value, the residual cholesterol inflammation

## Abstract

The residual cholesterol inflammation index (RCII) integrates residual cholesterol and C-reactive protein to assess for metabolic and inflammatory risk. We explored the link between the RCII and long-term risk of heart failure (HF) development in the United States. Data from the National Health and Nutrition Examination Survey were inspected, and 6027 participants aged 45 years and older were included. Natural log-transformed residual cholesterol inflammation index (ln RCII) values were used to classify participants into quartiles. The association between RCII and HF risk was examined, and potential covariates were controlled for using subgroup analyses, restricted cubic spline curves, subject work characteristic curve analysis techniques, and logistic regression models. The Q4 group (ln RCII > 14.82) was older, had an elevated body mass index, and a notably increased occurrence of diabetes, hypertension, and HF than the low ln RCII group (*P* < .05). Corrected logistic regression showed a higher HF in the Q4 group compared to the Q1 group (*P* < .05). HF risk increased by 36% (*P* < .001), 34% (*P* < .001), and 22% (*P* = .009) for every unit increase in ln RCII, indicating an independent positive correlation between RCII and HF risk. Restricted cubic spline regression confirmed a linear relationship (nonlinear *P* > .05). The receiver operating characteristic curve area under the curve was 0.59 (*P* < .05). Subgroup analyses revealed significant moderating associations for smoking status (interaction *P* = .034), stronger predictive validity of ln RCII among nonsmokers (*P* = .0014), no significant associations among smokers (*P* = .2375), and consistent positive associations for other subgroups. Elevated RCII levels are predictive of HF risk and are linked to an increased risk of HF in middle-aged and older populations.

## 1. Introduction

Population aging is a crucial factor driving the occurrence of cardiovascular diseases (CVD), thus presenting substantial public health challenges.^[1,2]^ CVD is the chief cause of mortality globally, with ischemic heart disease – a type of CVD – accounting for approximately 9 million deaths.^[3]^ Major risk factors, including hypertension, dyslipidemia, smoking, and chronic inflammation, are substantially linked to the morbidity and mortality rates associated with CVD.^[4-8]^ Heart failure (HF) is a serious condition and the final common pathway for CVD. It is caused by impaired ventricular filling or ejection, usually due to myocardial injury or structural changes, resulting in reduced cardiac output and systemic symptoms.^[9]^ The prevalence of both conditions increases significantly, posing a significant public health challenge that jeopardizes the well-being of middle-aged and older individuals. Early identification and management of risk factors associated with these conditions are crucial for reducing mortality in the aging demographic.

Residual cholesterol (RC) is an emerging indicator of abnormalities in lipid metabolism. It is obtained by subtracting high-density lipoprotein cholesterol (HDL-C) and low-density lipoprotein cholesterol (LDL-C) from total cholesterol (TC), resulting in the cholesterol content of triglyceride-rich lipoproteins.^[10,11]^ This can be indicative of potential pathological conditions present, such as atherosclerosis, cardiovascular events, and a variety of adverse health outcomes (like cancer and diabetes mellitus).^[12-17]^ Multiple pathways, including oxidative stress, lipid deposition, and the inflammatory response, are involved in the causative mechanism of RC.^[14,18]^ Inflammation is a key factor in both the development and progression of CVDs. Elevated C-reactive protein (CRP) is a classic inflammatory marker, and its measurement can determine the presence of CVDs such as HF, coronary heart disease, and stroke.^[19-22]^

The integration of inflammatory and metabolic markers to maximize cardiovascular risk assessment has gained significant interest in current research. By innovatively combining the inflammatory status and lipid metabolism disorders,^[23]^ the RCII – which is generated by multiplying RC by CRP – offers a fresh viewpoint on comprehensive cardiovascular risk assessment. While research has examined the link between RCII and stroke,^[24]^ its use in predicting HF risk needs further investigation, particularly regarding long-term risk assessment in large populations.

This study utilized nationally representative data from the National Health and Nutrition Examination Survey (NHANES) to examine the link between RCII and the risk of HF in middle-aged individuals and the elderly. The research aimed to systematically evaluate the predictive efficacy of RCII in determining HF and to investigate potential interactions that may enhance cardiovascular health management strategies for these populations.

## 2. Materials and methods

### 2.1. Study design and subjects

Data from the NHANES were used, and patients with HF from 1999 to 2010 were selected for the study. The data collected during the 1999 to 2010 cycle included measurements of TC, HDL-C, LDL-C, and CRP, which facilitated the calculation of RCII; however, the absence of CRP data in the 2011 to 2014 cycle resulted in its exclusion. Furthermore, the 2015 to 2018 cycle, which utilized high-sensitivity C-reactive protein, had inconsistencies in assay type, reduced follow-up durations (median 36 months), and a lower number of events; similarly, these restricted its comparability and statistical efficacy. Consequently, the analysis was limited to data collected from 1999 to 2010. NHANES is a program conducted by the National Center for Health Statistics (NCHS) and the Centers for Disease Control and Prevention (CDC). It evaluates the nutritional and health status of the US noninstitutionalized population based on a sophisticated, multistage stratified probability sampling method that targets specific counties, neighborhoods, households, and individuals within those households. Professionals trained by NCHS conduct in-home interviews with participants and undergo a comprehensive physical examination at a mobile examination center (MEC) that includes the collection of blood and urine. Comprehensive details regarding the implementation of the study have been made available at https://www.cdc.gov/nchs/nhanes/index.html.

**Figure 1.**
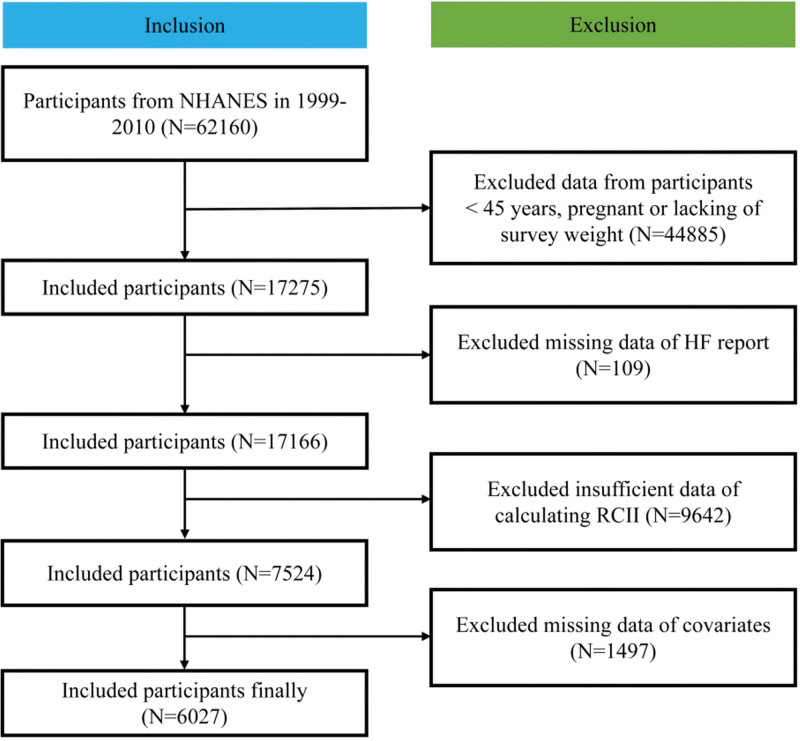
Flowchart illustrating the inclusion and exclusion criteria for study populations. HF = heart failure, NHANES = National Health and Nutrition Examination Survey, RCII = residual cholesterol inflammation index.

A total of 62,160 NHANES participants were initially included. Exclusion criteria were: 44,885 participants younger than 45 years, pregnant, or lacking survey weights; 109 participants with missing HF report data; 9642 participants with insufficient RCII data; and 1497 participants with missing data on covariates. Consequently, 6027 participants were eligible for the study, as shown in Figure 1.

### 2.2. Calculation of RC and RCII

NHANES healthcare professionals use enzyme colorimetric assays to determine lipid levels, like triglycerides, LDL-C, HDL-C, and TC, and immunoturbidimetric assays to determine CRP (mg/dL). The formula for calculating the RC is: RC (mg/dL) = TC − (HDL-C + LDL-C), and RCII is equal to = RC (mg/dL) × CRP (mg/dL).^[23]^

### 2.3. Diagnosis of heart failure

HF diagnosis: according to the NHANES Multiple Choice Questionnaire (MCQ160B) inquiry, “Has a doctor or other health professional ever told you that you have congestive heart failure?,” individuals who responded with a “yes” were categorized as having HF.^[25]^

### 2.4. Covariates

Potential covariates were identified in 3 areas: sociodemographic factors, lifestyle behavioral variables, and chronic disease. The sociodemographic characteristics examined included sex (female or male), age subgroups (40–59 and ≥60 years), race (Mexican American, other Hispanic, non-Hispanic White, non-Hispanic Black, and other races including multiracial mix), educational attainment (less than high school, high school graduate/general equivalency certificate or equivalent, and high school or higher), and the poverty-to-income ratio (PIR; which measures income relative to the federal poverty level, and accounts for factors such as economic inflation and family size). Marital status was grouped into 3 classes: married or cohabitating individuals, widowed, divorced, or separated individuals, and individuals who have never been married. Behavioral variables were divided into 3 categories: body mass index (BMI), smoking status, and drinking status, with BMI determined as BMI = weight (kg)/height (m^2^). The question “Ever smoked more than 100 cigarettes” was utilized in classifying people as smokers or never smokers, and the question “Drinking more than 12 drinks per year” was utilized in classifying people as drinkers or never drinkers.^[26]^ Chronic conditions were classified into diabetes mellitus or hypertension. Diabetes mellitus was defined as having fasting blood glucose levels ≥ 126 mg/dL, glycosylated hemoglobin levels ≥ 6.5 %, using antidiabetic medication use or a medical history,^[27]^ and hypertension was defined as having a systolic blood pressure ≥ 140 mm Hg, a diastolic blood pressure ≥ 90 mm Hg, using antihypertensive medications, or a reported medical history.^[28]^ Uric acid (UA) and serum creatinine (SCr) results were obtained by laboratory tests.

### 2.5. Statistical analysis

NHANES has an intricate multistage sampling design. Consequently, appropriate survey weights were utilized to represent the US population.^[29]^ We compared the distributions of several variables in 4 groups divided by RCII quartiles. Weighted *t* tests were employed for the analysis of continuous variables, which were reported as weighted means and standard deviations (SD). Weighted chi-square analysis was used to examine categorical variables, which were reported as numbers and weighted proportions.^[30]^ We applied a natural logarithm (Ln) transformation to the RCII to reduce the skewness.^[31]^ First, a weighted logistic regression model was utilized to evaluate the link between RCII and HF risk. Three models were selected utilizing RCII as a continuous variable: model 1 was unadjusted; model 2 was adjusted for demographic factors (age, gender, race, education level, PIR, and marital status); and model 3 built upon model 2, was further adjusted for smoking status, drinking status, diabetes, hypertension, UA, and serum creatinine (SCr).^[32]^ A restricted cubic spline (RCS) regression model was constructed to evaluate potential nonlinear relationships.^[33]^ To evaluate the diagnostic efficacy of the RCII, we used subject operating characteristic curve (ROC) analysis, by plotting the relationship between sensitivity (Sensitivity) and specificity (1 − Specificity), and using the cutoff value and area under the curve (AUC) to quantify its discriminatory ability.^[34]^ To determine the interaction effect of RCII with covariates on HF risk, we also conducted subgroup analyses. Likelihood ratio tests were used to evaluate the interaction effect.^[35]^ R (version 4.4.1; Vienna, Austria) was employed for all analyses, and a 2-sided *P* value < .05 was regarded statistically significant.

## 3. Results

### 3.1. Characteristics of the study population

The clinical characteristics of the participant scores are shown in Table 1. The study involved 6027 participants, who were classified into quartile groups according to natural log-transformed residual cholesterol inflammation index (ln RCII): Q1 (<2.38, n = 1522), Q2 (2.38–5.94, n = 1496), Q3 (5.94–14.82, n = 1506), and Q4 (>14.82, n = 1503). Baseline characteristics showed that the Q4 group was older, had a smaller proportion of males, lower levels of education and income, increased BMI, lower likelihood of being non-Hispanic White, fewer married or cohabitating individuals, higher likelihood of being a smoker, lower likelihood of being an alcoholic, and an elevated incidence of diabetes, hypertension, SCr, UA levels, and HF, compared to the group with lower ln RCII (all *P* < .05).

**Table 1:** Characteristics of participants grouped by ln RCII quartiles, NHANES 1999–2010.

Variables	Total	Quartiles of ln RCII				*P*
		Q1 (<2.38)	Q2 (2.38, 5.94)	Q3 (5.94, 14.82)	Q4 (>14.82)	
n	6027	1522	1496	1506	1503	
Age, n (%)						<.001
40–59	2436 (56.6)	726 (64.3)	573 (54.3)	550 (51.3)	587 (55.3)	
≥60	3591 (43.4)	796 (35.7)	923 (45.7)	956 (48.7)	916 (44.7)	
Gender, n (%)						<.001
Male	3024 (47.8)	840 (52.3)	814 (52.3)	761 (47.9)	609 (37.2)	
Female	3003 (52.2)	682 (47.7)	682 (47.7)	745 (52.1)	894 (62.8)	
Race, n (%)						.001
Mexican American	1032 (4.6)	173 (3.0)	259 (4.6)	284 (5.3)	316 (5.7)	
Other Hispanic	342 (3.1)	87 (2.6)	82 (2.9)	94 (4.0)	79 (3.2)	
Non-Hispanic White	3429 (79.6)	917 (80.0)	880 (81.8)	821 (77.7)	811 (78.6)	
Non-Hispanic Black	1040 (8.6)	275 (8.3)	233 (7.4)	268 (9.5)	264 (9.3)	
Other race – including multiracial	184 (4.1)	70 (6.1)	42 (3.3)	39 (3.5)	33 (3.2)	
Education, n (%)						<.001
Less than high school	1912 (19.6)	362 (14.4)	468 (19.4)	540 (21.7)	542 (24.1)	
High school graduate/GED or equivalent	1413 (25.8)	325 (21.1)	367 (27.6)	354 (27.4)	367 (27.9)	
Higher than high school	2702 (54.6)	835 (64.6)	661 (53.0)	612 (50.9)	594 (48.0)	
PIR, n (%)						<.001
≤1.3	1542 (15.7)	297 (11.4)	340 (13.8)	411 (17.6)	494 (20.8)	
1.3–3.5	2370 (35.4)	559 (31.0)	621 (37.8)	617 (38.0)	573 (35.5)	
>3.5	2115 (48.9)	666 (57.6)	535 (48.5)	478 (44.4)	436 (43.7)	
Marital status, n (%)						.002
Married/living with partner	3844 (70.1)	1033 (73.7)	976 (71.6)	949 (69.0)	886 (65.1)	
Widowed/divorced/separated	1872 (25.5)	406 (21.9)	454 (24.9)	478 (26.3)	534 (29.4)	
Never married	311 (4.5)	83 (4.4)	66 (3.5)	79 (4.6)	83 (5.5)	
BMI, n (%)						<.001
<25 kg/m^2^	1629 (28.5)	707 (50.4)	399 (27.4)	289 (18.0)	234 (14.0)	
25–30 kg/m^2^	2233 (36.4)	561 (35.3)	635 (42.7)	593 (38.6)	444 (28.7)	
≥30 kg/m^2^	2165 (35.1)	254 (14.2)	462 (29.9)	624 (43.4)	825 (57.3)	
Smoking status, n (%)						<.001
Smokers	3207 (53.3)	745 (48.9)	781 (52.2)	839 (54.1)	842 (59.0)	
Non smokers	2820 (46.7)	777 (51.1)	715 (47.8)	667 (45.9)	661 (41.0)	
Drinking status, n (%)						<.001
Drinkers	4059 (71.0)	1099 (76.1)	1032 (72.7)	996 (67.9)	932 (66.3)	
Non drinkers	1968 (29.0)	423 (23.9)	464 (27.3)	510 (32.1)	571 (33.7)	
Diabetes, n (%)						<.001
Yes	1353 (17.2)	254 (12.1)	304 (15.5)	361 (18.9)	434 (23.7)	
No	4674 (82.8)	1268 (87.9)	1192 (84.5)	1145 (81.1)	1069 (76.3)	
Hypertension, n (%)						<.001
Yes	3610 (52.8)	762 (41.1)	883 (52.5)	955 (57.3)	1010 (62.6)	
No	2417 (47.2)	760 (58.9)	613 (47.5)	551 (42.7)	493 (37.4)	
SCr, mg/dL	0.91 (0.38)	0.90 (0.23)	0.92 (0.34)	0.92 (0.41)	0.92 (0.50)	.077
UA, mg/dL	5.61 (1.38)	5.17 (1.28)	5.62 (1.31)	5.80 (1.36)	5.94 (1.45)	<.001
HF, n (%)						<.001
Yes	284 (3.7)	44 (2.1)	68 (3.1)	69 (4.0)	103 (5.9)	
No	5743 (96.3)	1478 (97.9)	1428 (96.9)	1437 (96.0)	1400 (94.1)	

BMI = body mass index, CI = confidence interval, GED = general educational development, HF = heart failure, ln RCII = natural log-transformed residual cholesterol inflammation index, NHANES = National Health and Nutrition Examination Survey, OR = odds ratio, PIR = poverty-to-income ratio, SCr = serum creatinine, UA = uric acid.

### 3.2. Association between ln RCII and HF

Weighted logistic regression (Table 2) showed that higher ln RCII levels were substantially linked to increased risk of HF in the unadjusted model 1. The risk of HF was 81% higher in the Q4 group than in the Q1 group in the fully adjusted model (model 3; odds ratio [OR] = 1.81, 95% confidence interval [CI]: 1.02–3.21, *P* < .05), indicating a significant trend of increasing risk across quartiles (*P* < .05 for trend). The risk of HF rose by 36% (*P* < .05 for trend) for each unit increase in In RCII in the unadjusted, adjusted-demographics, and fully adjusted models. HF risk increased by 36% (OR = 1.36, 95% CI: 1.22–1.52, *P* < .001), 34% (OR = 1.34, 95% CI: 1.19–1.52, *P* < .001), and 22% (OR = 1.22, 95% CI: 1.05–1.42, *P* = .009), showing an independent positive link between RCII and HF risk.

**Table 2:** Correlation between ln RCII and HF in weighted logistic regression models.

	Model 1	*P*	Model 2	*P*	Model 3	*P*
	OR (95% CI)		OR (95% CI)		OR (95% CI)	
Continuous	1.36 (1.22, 1.52)	<.001	1.34 (1.19, 1.52)	<.001	1.22 (1.05, 1.42)	.009
Q1	Reference				Reference	
Q2	1.47 (0.95, 2.29)	.086	1.22 (0.78, 1.92)	.375	1.03 (0.64, 1.66)	.907
Q3	1.88 (1.15, 3.09)	.013	1.5 (0.89, 2.51)	.124	1.17 (0.66, 2.08)	.576
Q4	2.87 (1.83, 4.50)	<.001	2.6 (1.60, 4.22)	<.001	1.81 (1.02, 3.21)	.043
*P* for trend	<.001		<.001		.029	

Model 1 was not controlled for any covariates.

CI = confidence interval, OR = odds ratio, HF = heart failure, ln RCII = natural log-transformed residual cholesterol inflammation index.

**Figure 2.**
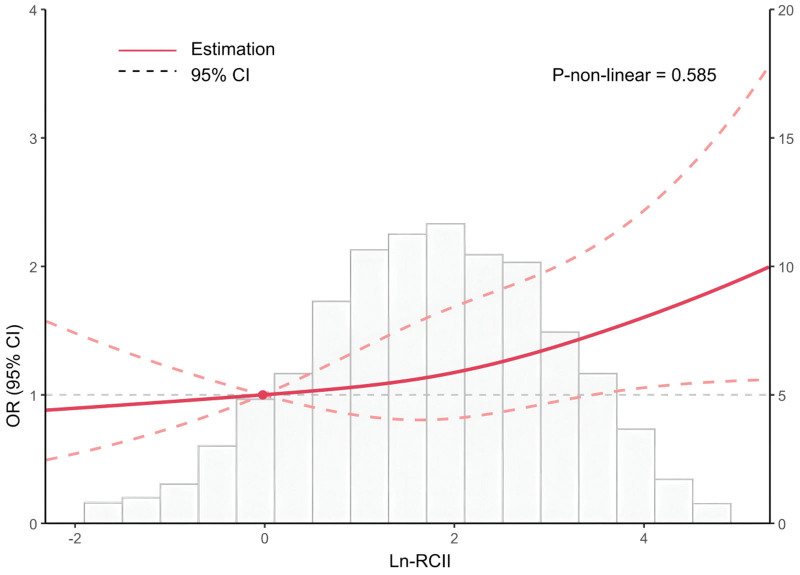
Correlation between ln RCII and HF in the RCS regression model. Models were controlled for age, sex, ethnicity, education, PIR, marital status, BMI, smoking status, drinking status, diabetes, hypertension, SCr, and UA. BMI = body mass index, CI = confidence interval, HF = heart failure, ln RCII = natural log-transformed residual cholesterol inflammation index, OR = odds ratio, PIR = poverty-to-income ratio, RCS = restricted cubic spline, SCr = serum creatinine, UA = uric acid.

Subsequently, we developed an RCS regression model to investigate the nonlinear link between ln RCII and HF (Fig. 2). The model showed that ln RCII and HF risk were linearly and positively correlated after accounting for all covariates (all nonlinear *P* values > .05).

### 3.3. ROC curve and subgroup analysis

**Figure 3.**
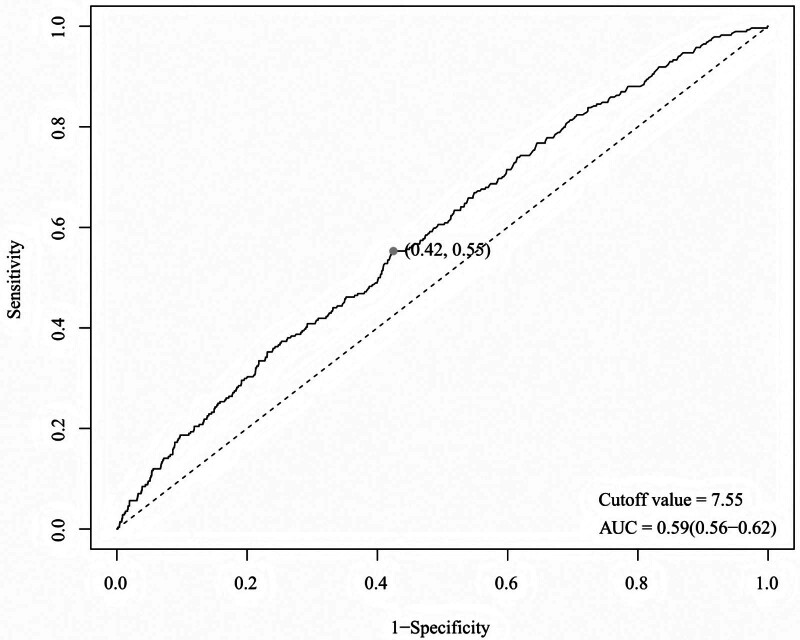
Predictive efficacy of ln RCII for HF in the ROC model. AUC = area under the curve, HF = heart failure, ln RCII = natural log-transformed residual cholesterol inflammation index, ROC = operating characteristic curve.

The ROC curve was employed to evaluate the predictive efficacy of ln RCII for HF, as illustrated in Figure 3. The AUC of ln RCII (OR = 0.59, 95% CI: 0.56–0.62) demonstrated a moderate predictive efficacy for HF risk, showing a slight improvement over the randomized classification (AUC = 0.5). However, the overall predictive effectiveness was average, and the findings were statistically significant (*P* < .05), excluding chance of association. The Jordon index obtained the ideal cutoff value of 7.55, which was used as a critical value for identifying HF risk. This allowed the model to achieve a relatively optimal balance between sensitivity (the capacity to accurately identify HF patients) and specificity (the capacity to accurately exclude individuals without HF).

Subgroup analyses (Table 3) showed a consistent positive link between ln RCII and the risk of HF. Notably, our analysis revealed that the smoking status strongly influenced the link between ln RCII and HF (interaction *P* value < .05). The predictive validity of ln RCII for HF risk was found to be more pronounced in nonsmokers (OR = 1.39, 95% CI: 1.14–1.70), while the correlation was weaker and not statistically significant in smokers (OR = 1.11, 95% CI: 0.93–1.33).

**Table 3:** Subgroup analysis of ln RCII and HF correlation.

Characteristic	OR (95% CI)	*P* value	*P* for interaction
Age, n (%)			.779
40–59	1.38 (0.98, 1.94)	.0652	
≥60	1.19 (1.03, 1.37)	.0211	
Gender, n (%)			.865
Male	1.21 (1.01, 1.46)	.0411	
Female	1.24 (1.01, 1.51)	.0395	
Race, n (%)			.406
Mexican American	1.02 (0.68, 1.53)	.9122	
Other Hispanic	2.61 (0.92, 7.41)	.0743	
Non-Hispanic White	1.22 (1.02, 1.45)	.0299	
Non-Hispanic Black	1.08 (0.79, 1.49)	.6303	
Other race – including multiracial	3.03 (0.85, 10.78)	.0911	
Education, n (%)			.835
Less than high school	1.21 (0.99, 1.47)	.0609	
High school graduate/GED or equivalent	1.31 (0.98, 1.76)	.0711	
Higher than high school	1.19 (0.96, 1.49)	.1182	
PIR, n (%)			.925
≤1.3	1.17 (0.94, 1.46)	.1545	
1.3–3.5	1.29 (1.08, 1.55)	.0074	
>3.5	1.14 (0.81, 1.62)	.4525	
Marital status, n (%)			.67
Married/living with partner	1.30 (1.04, 1.62)	.0242	
Widowed/divorced/separated	1.13 (0.93, 1.36)	.2196	
Never married	1.10 (0.59, 2.04)	.7613	
BMI, n (%)			.68
<25 kg/m^2^	1.39 (1.10, 1.75)	.0068	
25–30 kg/m^2^	1.21 (0.93, 1.57)	.151	
≥30 kg/m^2^	1.17 (0.94, 1.45)	.1659	
Smoking status, n (%)			.034
Smokers	1.11 (0.93, 1.33)	.2375	
Non smokers	1.39 (1.14, 1.70)	.0014	
Drinking status, n (%)			.411
Drinkers	1.18 (1.01, 1.39)	.0413	
Non drinkers	1.31 (1.04, 1.65)	.0234	
Diabetes, n (%)			.884
Yes	1.20 (0.94, 1.52)	.1493	
No	1.24 (1.03, 1.49)	.0238	
Hypertension, n (%)			.151
Yes	1.17 (1.01, 1.36)	.0419	
No	1.39 (1.00, 1.93)	.0558	

Models were controlled for age, sex, ethnicity, education, PIR, marital status, BMI, smoking status, drinking status, diabetes, hypertension, SCr, and UA.

BMI = body mass index, CI = confidence interval, GED = general educational development, HF = heart failure, ln RCII = natural log-transformed residual cholesterol inflammation index, OR = odds ratio, PIR = poverty-to-income ratio, SCr = serum creatinine, UA = uric acid.

## 4. Discussion

Nationally representative data from NHANES were analyzed; a strong link between ln RCII and HF risk was elucidated. This provides new evidence for integrating metabolic and inflammatory indicators to predict CVD. According to the baseline data, ln RCII was positively and independently related to the risk of HF. A linear positive link between ln RCII and HF was revealed by RCS analysis. ROC curves indicated that ln RCII could predict the risk of HF to an extent. A stronger link between ln RCII and nonsmokers was revealed by subgroup analyses. Studies that specifically evaluate the effect of integrated metabolic-inflammatory markers like RCII on the prognosis of HF are limited. This makes the findings from our research a valuable contribution to the field, as they offer a more comprehensive discussion regarding the molecular mechanisms underlying the activation of residual cholesterol inflammation during the onset and progression of HF. As a result, a strong foundation can be established for the development of personalized precision treatment. Nutritional intervention is a simple and practical way to address and regulate metabolic dysregulation and inflammatory states; therefore, it should be further investigated for its involvement in reducing the risk of HF in certain populations, like individuals with high levels of RCII. This approach may open new and innovative treatment and prevention methods that are cost-effective and easily implementable.

HF is a complex syndrome marked by compromised cardiac function. Its pathogenesis involves multiple pathways such as neuroendocrine activation, oxidative stress, apoptosis, metabolic remodeling, and chronic inflammation.^[36-40]^ In recent years, the interaction between metabolism and inflammation, referred to as the “metabolism-inflammation axis,” has emerged as a central driver in the development of HF,^[41,42]^ with various parameters of RCII being closely linked to the pathogenesis of HF.^[43-46]^ In the field of lipid metabolism, HDL-C exerts cardioprotective effects through certain mechanisms, including endothelial protection, anti-inflammatory regulation, and reverse cholesterol transport.^[47]^ Epidemiological research reveals that every 1 mg/dL increase in HDL-C lowers the risk of CVD by 2% to 3%, and even after controlling for confounding variables like age and blood pressure, the protective effect remains significant.^[48]^ However, low HDL-C (<40 mg/dL) has been linked to coronary heart disease, a major cause of HF. It is worth noting that research shows that the functional quality of HDL-C, such as cholesterol efflux capacity, may be more beneficial than concentration alone. This is because dysfunctional HDL particles lose their anti-inflammatory qualities during myocardial remodeling and fibrosis, which exacerbates oxidative stress in cardiomyocytes and generates abnormal macrophage polarization.^[49-51]^ HDL-C elevation has long been considered a target for intervention; however, paradoxical observations in clinical practice were made when significant elevations in HDL-C levels (30–70%) through niacin and cholesteryl ester transfer protein inhibitors did not consistently translate into a corresponding reduction in cardiovascular events.^[50]^ This phenomenon suggests that the “functional heterogeneity” of HDL-C may influence therapeutic efficacy. For example, in diabetic patients, HDL particles with altered ApoA-I conformation due to glycosylation modifications may have a reduced antioxidant capacity, thus exacerbating myocardial mitochondrial dysfunction.^[52-54]^

RC, a marker of residual atherogenic lipoproteins, is frequently underutilized in clinical practice despite its association with increased inflammation and CVD risk. While RC is linked to HF, the relationship shows some inconsistencies and contradictions.^[55]^ While RC is linked to HF, its association with HF shows significant heterogeneity and contradictions. Nevertheless, data from a large cohort have revealed that increased RC levels were substantially linked to an increased risk of CVD.^[56]^ Liu discovered that HF risk was linked to both the duration of RC accumulation and long-term exposure to it, with early RC accumulation increasing risk relative to late accumulation.^[57]^ In hypertensive HF patients, RC was linked to left ventricular remodeling; patients with left ventricular hypertrophy had higher RC values, and those with hypertensive HF who had higher RC were at higher risk of dying from cardiovascular causes.^[58]^ Conversely, the ChinaHEART study demonstrated that in HF patients, lower RC levels were linked to higher mortality,^[59]^ suggesting that RC may affect the outcome of different HF subtypes through a dual mechanism (proinflammatory damage and metabolic compensation). The “double-edged sword” effect of RC may be due to the following factors: proinflammatory (RC is susceptible to oxidative modification to form oxidized phospholipids, which activate the TLR4/MyD88/NF-κB pathway to induce myocardial apoptosis)^[60,61]^ and metabolic compensation (RC is catabolized into free fatty acids to replace glucose for energy in advanced HF).^[62,63]^ RCII integrates the lipid metabolism disorder signaling of RC with the chronic inflammation signaling of CRP. It advances atherosclerosis by facilitating lipid buildup, inducing oxidative stress, and causing endothelial dysfunction^[64-67]^; furthermore, CRP may exacerbate these effects by promoting vascular injury, monocyte recruitment, and inflammation-driven angiogenesis.^[68,69]^ Thus resulting in synergistic effects.

Notably, inflammation-driven physiological stress is a key factor in the development of HF, as well as a significant predictor of patient prognosis. Several investigations using numerous biomarkers have been conducted to validate these findings in clinical analyses. The nutritional status of an individual can influence the inflammatory response of the body. Due to this interaction, nutritional deficiencies may aggravate systemic inflammation, resulting in an impaired myocardial repair system. Furthermore, a dysregulated inflammatory response can disrupt the body’s metabolic function, reducing the availability of nutrients, thus creating a vicious cycle.^[70]^ Therefore, the evaluation of HF prognosis is significantly improved by integrating indicators from both areas. For example, the CRP to albumin ratio and the prognosis nutrition index are useful indicators of long-term mortality in patients with HF with reduced ejection fraction (HFrEF) who are treated with implantable cardioverter-defibrillators (ICDs). This provides simple and efficient applications for clinical prognosis assessment.^[71,72]^ Furthermore, according to a comprehensive analysis involving 52 prospective studies and 2,46,669 participants who had no previous history of CVD, CRP and fibrinogen improve cardiovascular risk classification beyond traditional factors like age, blood pressure, and lipids, demonstrating particularly significant stratification value for intermediate-risk populations.^[73]^ Additionally, a meta-analysis found that a number of easily available inflammation-nutrition integrated indicators exhibit exceptional prognostic prediction value for HF. It was discovered that there was a substantial correlation between the standard inflammatory marker, the neutrophil-to-lymphocyte ratio (NLR), and the risk of long-term all-cause mortality in patients with HFrEF.^[74]^ Dynamic monitoring of NLR levels before hospital admission and discharge further enhances predictive efficacy. Predischarge NLR levels serve as an independent predictor of long-term all-cause mortality following the discharge of HFrEF patients. Additionally, the combination of both preadmission and predischarge NLR measurements enhances predictive accuracy. The evaluation of nutrition-related indices like the control of nutritional status (CONUT) score, which combines serum albumin, cholesterol, and lymphocyte count, shows good predictive accuracy for in-hospital mortality and independently predicts adverse clinical outcomes in patients with acute decompensated HF. Moreover, meta-analyses validate the significant positive correlation between elevated CONUT scores and increased all-cause mortality risk in HF patients. It has been determined that initiating nutritional supplementation within 48 hours of admission effectively reduces mortality risk.^[75]^ These findings show a similar biological significance to the inflammation-related prognostic risks identified in cases of pericarditis, collectively suggesting a strong link between systemic inflammatory conditions, nutritional deficiencies, myocardial inflammation, and hemodynamic injury. We can therefore surmise that nutritional deficiencies impair the restoration of cardiac contractile function, whereas chronic inflammatory responses aggravate myocardial cell apoptosis. The synergistic effects of both ultimately drive HF progression and elevate mortality risk. Drawing from the pathophysiological mechanism mediated by nutrition and inflammation, previous studies have shown the considerable benefit of integrated CRP and RC research in predicting the risk of atherosclerotic CVD.^[76,77]^ This study further expands the application of this integrated indicator to the assessment of the potential risk of HF, providing a novel metabolic-inflammatory co-regulation perspective for HF risk stratification.

The RCS regression model confirmed that ln RCII was linearly and positively correlated with the risk of HF, indicating that the risk of HF increased as ln RCII levels rose. This was in line with the trend observed in the logistic regression analysis. The ROC curves showed that ln RCII could moderately differentiate the risk of HF, suggesting its potential as an auxiliary predictive index. However, its standalone application has limited clinical value, and it should be combined with risk factors such as hypertension, diabetes mellitus, or specific biomarkers such as natriuretic peptides to optimize the risk stratification.^[78]^ Currently, risk stratification for complex CVDs has gradually incorporated predictive models based on machine learning (ML) and deep learning (DL). These advanced tools have demonstrated advantages over traditional risk scores, thereby enhancing decision-making in clinical practice. For instance, in a study by Cicek et al, a multimodal deep learning (mmDL) model integrating CT imaging and clinical data successfully achieved precise short-term mortality prediction for patients with acute pulmonary embolism, significantly outperforming the traditional pulmonary embolism severity index (PESI).^[79]^ A separate published study introduced the AI-driven CLASHED risk prediction model specifically designed for elderly patients undergoing non-elective surgery.^[80]^ By using easily accessible parameters like creatinine and lymphocyte count, this model accurately assessed the risk of myocardial injury not related to cardiac surgery (MINS) postoperatively, demonstrating a predictive performance significantly superior to the revised cardiac risk index (RCRI). Integrating biomarkers like RCII, which consolidate metabolic and inflammatory information, with AI/ML models facilitates the integration of multidimensional clinical, imaging, and laboratory data. This approach also has the potential to further improve the accuracy and clinical application of HF risk stratification. These findings link the analyses observed in this study to the larger field of inflammation-mediated prognostic medicine and AI-assisted diagnosis/treatment. It also provides crucial guidance for future clinical practice by integrating advanced detection markers with modern predictive methods, offering new insights for applying the “metabolic-inflammatory co-regulation” theory and developing low-cost comprehensive risk indicators.

Subgroup analyses showed that smoking status significantly influenced the link between ln RCII and HF risk, with the predictive validity of ln RCII being significantly stronger in nonsmokers than in smokers. This result is consistent with previous studies on the disruptive effects of smoking on inflammatory pathways. Smoking activates the NF-κB pathway, induces the release of proinflammatory factors such as IL-6 and TNF-α,^[81,82]^ and facilitates a strong proinflammatory state independent of RCII, which masks the link between RCII and HF. In nonsmokers, the “metabolic-inflammatory” synergy of RCII is more evident, suggesting that RCII more accurately reflects the potential risk of HF in the absence of smoking-associated inflammatory disturbances. Furthermore, several studies have shown that HF development is substantially impacted by smoking^[83-85]^ and is also a chief driver of systolic dysfunction, LV hypertrophy, and HF hospitalization,^[86]^ all of which are in line with the findings of this investigation. However, the subgroup analysis only categorized smoking status into “smokers” and “nonsmokers” without distinguishing current smoking level or length of cessation, which may affect the accurate assessment of the interaction. Future studies may further explore the effects of smoking intensity, duration of cessation, and more detailed ethnic stratification on the predictive efficacy of the RCII to optimize accurate stratification strategies for HF risk in middle-aged individuals and the elderly. This aligns closely with the central direction of personalized medicine. In current research, the focus of personalized medicine is to expand its application from disease treatment to prevention, enabling targeted interventions through precise risk stratification. Key practices include polygenic risk scoring (PRS) and rehabilitation genomics.^[87]^ This study is a crucial investigation that directly addresses the clinical significance of integrated metabolic-inflammatory markers to thoroughly evaluate the effect of RCII on HF prognosis. The linear relationship between RCII and the risk of HF, together with the regulatory effect of the smoking status, offers population-based evidence that reveals the pathophysiological mechanisms through which the “metabolic-inflammatory axis” drives the progression of HF. It also enables the identification of crucial regulatory factors involved in the disease advancement. This evidence serves as the core basis for developing individualized precision treatment strategies.

This research reveals the link between metabolic dysfunction, inflammation, and morbidity risk based on large-scale nationally representative data. The innovative integration of metabolic RC and inflammatory CRP metrics provides new perspectives for HF risk assessment. Subgroup analyses revealed the regulatory role of smoking status and strengthened the pathological mechanism of the “metabolism-inflammation axis” in HF. The study design was rigorous, and the conclusions were generalizable to the population. We demonstrated that the RCII could be applied in clinical practice as a useful and instructive tool for early intervention and mortality risk stratification.

## 5. Limitations

There are several limitations present in the study. First, this is a cross-sectional study, which means it only demonstrated a cross-sectional relationship between RCII and HF, without confirming the causality or long-term dynamic effects. This should be investigated in future longitudinal cohort studies. Second, the diagnosis of HF relied on questionnaire self-report rather than on objective indicators such as echocardiography or natriuretic peptide, which may have recall bias or underdiagnosis. Third, the calculation of RCII was derived from formula estimation rather than direct measurement of residual lipoprotein particles (such as ultracentrifugation), which could be limited by the error of the Friedewald formula at high triglyceride levels. The study did not include key indicators reflecting cardiac structure and function, such as left ventricular ejection fraction and N-terminal pro-B-type natriuretic peptide, which may affect the in-depth analysis of the pathophysiologic mechanisms of HF. In subgroup analyses, some subgroups (such as specific ethnicity or education level) had small sample sizes; therefore, the results of interactions should be interpreted cautiously. Finally, the smoking status was only categorized into “smokers” and “nonsmokers” without refining factors such as smoking intensity and length of cessation, which may underestimate the complex effects of smoking on the RCII-HF association. These limitations provide directions for follow-up studies, such as combining longitudinal data, objective diagnostic criteria, and precise molecular tests, to further clarify the clinical value of RCII in HF prediction.

## 6. Conclusion

Higher RCII levels are predictive of HF risk and are substantially linked to an increased risk of HF in middle-aged individuals and the elderly. The integration of metabolic and inflammatory markers enables a comprehensive assessment of mortality risk among middle-aged and older individuals in the United States. The RCII proves advantageous for clinical risk stratification, particularly in identifying individuals at high risk, due to its simple application and high predictive power. However, to confirm its practicality and validate its application in clinical practice, further verification in longer-term prospective studies and larger multiethnic cohorts is required.

## Acknowledgments

We are grateful to the NHANES staff and participants for their valuable contributions, particularly for providing data licenses. We extend special thanks to Professor Zhixi Hu for his invaluable contributions to the research ideas and funding support for this paper.

## Author contributions

**Data curation:** Ji Ouyang, Jiayuan Song.

**Conceptualization:** Jiayuan Song.

**Formal analysis:** Ji Ouyang, Jiayuan Song.

**Funding acquisition:** Zhixi Hu.

**Methodology:** Guicheng Liu.

**Resources:** Zhixi Hu.

**Supervision:** Zhixi Hu.

**Writing – original draft:** Ji Ouyang, Xingxing Zhang.

**Writing – review & editing:** Ji Ouyang, Jiayuan Song, Xingxing Zhang, Guicheng Liu.
